# Curing of Cellulose Hydrogels by UV Radiation for Mechanical Reinforcement

**DOI:** 10.3390/polym13142342

**Published:** 2021-07-17

**Authors:** Rodybeth Cruz-Medina, Daniel Alejandro Ayala-Hernández, Alejandro Vega-Rios, Erika Ivonne López-Martínez, Mónica Elvira Mendoza-Duarte, Anayansi Estrada-Monje, Erasto Armando Zaragoza-Contreras

**Affiliations:** 1 Centro de Investigación en Materiales Avanzados, SC, Department of Engineering and Materials Chemistry, Miguel de Cervantes No. 120, Complejo de Industrial Chihuahua, Chihuahua CP 31136, Mexico; rodybeth.cruz@cimav.edu.mx (R.C.-M.); AlejandroAyala45@hotmail.com (D.A.A.-H.); alejandro.vega@cimav.edu.mx (A.V.-R.); erika.lopez@cimav.edu.mx (E.I.L.-M.); monica.mendoza@cimav.edu.mx (M.E.M.-D.); 2 Centro de Innovación Aplicada en Tecnologías Competitivas, AC, Calle Omega No. 201, Industrial Delta, León, Guanajuato CP 37545, Mexico; aestrada@ciatec.mx

**Keywords:** cellulose, hydrogel, mechanical properties, photodegradation, UV radiation

## Abstract

The use of biomaterials as a replacement for thermoplastic polymers is an environmentally sound strategy. In this work, hydrogels of cellulose isolated from wheat husk were modified by UV irradiation (353 nm) to improve mechanical performance. The cellulose was dissolved with a solvent system *N,N*-dimethylacetamide/lithium chloride (DMAc/LiCl). Infrared spectroscopy showed that the peak height at 1016 cm^−1^, associated with the C–O bonds of the glycosidic ring, increases with irradiation time. It was determined that the increase in this signal is related to photodegradation, the product of a progressive increase in exposure to UV radiation. The viscoelastic behavior, determined by dynamic mechanical analysis and rotational rheometry, was taken as the most important parameter of this research, showing that the best results are recorded with 15 min of UV treatment. Therefore, at this time or less, the chemical crosslinking is predominant over the photodegradation, producing an increase in the modules, while with 20 min the photodegradation is such that the modules suffer a significant reduction.

## 1. Introduction

In response to the problems generated by the accumulation of plastics in the environment, modern requirements in materials science have stimulated the development of a great variety of biobased materials, in which cellulose and its derivatives play a leading role [1]. The most important reason for this is that cellulose is nature’s most abundant renewable polymeric biomaterial. Furthermore, to access sources rich in cellulose, it is only enough to resort to agro-industrial wastes, such as grain and seed crop residues.

The chemical and structural characteristics of cellulose are well known [2]. Due to its complex inter- and extra-molecular interactions, cellulose molecules are tightly bound through hydrogen bonds, being insoluble in conventional polar solvents such as water, alcohols, and amines, making it difficult to process into the desired shape. For decades, special solvent systems have been developed to dissolve cellulose so that it can be processed. Examples of these are urea/sodium hydroxide [3,4], ionic liquids [5,6], or *N*,*N*-dimethylacetamide (DMAc)/lithium chloride (LiCl) [7,8]. 

The DMAc/LiCl solvent system was first reported by McCormick [9]. The interaction of this solvent system with cellulose has been the subject of study since then, and although there is no total consensus, there are some interaction models [8,10,11]. The system DMAc/LiCl dissolves cellulose efficiently, the regeneration product being a gel swollen with water or hydrogel. Cellulose and its derivatives in the hydrogel form are very convenient since, in this state, these can be used in a wide variety of applications, e.g., controllable delivery [12], or medical applications [13].

A disadvantage of hydrogels, in general, is the poor mechanical strength [14]; therefore, it is of great interest to improve said performance to expand their usable margin towards applications that require more significant mechanical stress, such as in water treatment membranes. Physical and chemical methods can modify the mechanical properties of cellulose-based hydrogels. In the former, the physical interactions between the hydrophobic moieties of the components and the hydrogen bonds produce the crosslinking and can be considered a weak molecular association [15]. In the reinforcement by physical crosslinking, the addition of a second polymer in solution is also included, which provides properties to the hydrogel [16]. For the second method, crosslinking agents are used in order to react with the functional groups [17,18]; nonetheless, they have the disadvantage of leaving toxic reaction remnants, or sometimes the crosslinker is itself toxic [19]. 

Additionally, the treatment or curing of cellulose-based materials by high-energy radiation (gamma rays, electron beam, or ultraviolet radiation) [20,21], has made it possible to carry out chemical modifications due to crosslinking [22] and other kinds of reactions [23], which have substantially improved their mechanical properties [24]. Therefore, it can be mentioned, as advantages, that it does not use crosslinking agents, does not generate waste, and it simultaneously produces sterilization during irradiation [25]. However, it has the drawback that it can cause degradation if exposure is not carefully controlled [22].

In this work, we report for the first time, the curing of cellulose isolated from the wheat husk in a solution of DMAc/LiCl, employing UV radiation (354 nm) to improve hydrogel mechanical performance. The study is based on the viscoelastic behavior of the hydrogel as a function of UV radiation time, analyzed by dynamic mechanical analysis and rotational rheometry. The most critical changes in cellulose due to high energy radiation are crosslinking and photochemical degradation. The techniques report these effects like changes in the behavior of the modules. Wheat husk was selected as a source of cellulose because it is a residue widely available throughout the world, to which little technological development has been applied. The cellulose curing was performed before the regeneration so that the cellulose molecules were in solution to improve crosslinking. It is worth noting that for the curing of the hydrogels, no crosslinking agent or other polymer was used to generate the 3D network as in other reported systems. Only cellulose and UV radiation, as the trigger for the crosslinking, were used. The obtained hydrogels were also characterized by infrared spectroscopy, thermogravimetry, and X-ray diffraction.

## 2. Materials and Methods

### 2.1. Materials

The cellulose was extracted from wheat husk supplied by a local producer in Chihuahua, Chih., Mexico. Acetic acid (Fermont, Monterrey, Nuevo León, México, 99%), formic acid (J.T. Baker, Phillipsburg, NJ, USA, 88%), sodium hypochlorite solution (Merck, Sigma-Aldrich, Saint Louis, MO, USA, 10–15%), hydrogen peroxide (Golden Bell, Orange, CA, USA, 30%), *N,N*-dimethylacetamide (Merck, Sigma-Aldrich, Saint Louis, MO, USA, >99%), and lithium chloride (Merck, Sigma-Aldrich, Saint Louis, MO, USA, >99%) were used as received.

### 2.2. Cellulose Isolation

To isolate the wheat husk cellulose (WHC), in the first step, the organosolv method was used [26], which is described as follows: a solution (1000 mL) of acetic acid (60 vol%), formic acid (30 vol%), and water (10 vol%) was prepared. The solution was heated to 85 °C and then 40 g of wheat husk, previously washed with distilled water to remove traces of dust, was added to the reactor, maintaining heating and with gentle magnetic stirring for 4 h. Then, the reactor was allowed to cool to room temperature and the mixture vacuum filtered to recover the solid, which was washed with abundant distilled water to a neutral pH. Finally, the solid was dried in an oven at 80 °C for 14 h.

In the next step, the cellulose was brought to a first bleaching treatment with a 13% sodium hypochlorite (NaClO) solution. In addition, the ratio of cellulose to NaClO was 1:25 wt/vol (1 g: 25 mL). The system was kept under gentle magnetic stirring at room temperature for 2 h. Afterward, the cellulose fiber was recovered by filtration and washed with distilled water to a neutral pH. The fiber was recovered and dried in an oven at 80 °C for 14 h.

In the third stage, a second bleaching treatment was performed, using a 35% hydrogen peroxide, at cellulose to peroxide ratio 1:25 wt/vol. First, the peroxide solution was heated to 90 °C; then, the pre-bleached cellulose fiber was added, and the system was kept under gentle magnetic stirring for 2 h. Subsequently, the fiber was recovered by filtration and submitted to washing with distilled water to a neutral pH. Finally, the cellulose was dried in an oven at 80 °C for 14 h.

### 2.3. Cellulose Fiber Dissolution in DMAc/LiCl System

Cellulose dissolution was performed based on the reported methodology [27]. A total of 100 mL of *N,N*-dimethylacetamide (DMAc) was heated at 120 °C for 60 min to evaporate the absorbed water. Next, the solvent was allowed to cool to 60 °C, and then 8 g of lithium chloride (LiCl) was added, maintaining gentle magnetic stirring until dissolution. Subsequently, 2 g of dry cellulose was added, keeping the temperature and magnetic stirring until fiber dissolution, which takes 6 days on average.

### 2.4. Cellulose Recovering and UV Curing

For cellulose curing with UV radiation and subsequent regeneration, the following procedure was achieved: 4 mL of the cellulose solution was placed in a Petri dish; then, the solution was exposed to a 365 nm wavelength UV radiation curing treatment using a lamp (Model ENF-260C, Spectroline, Melville, NY, USA). The distance between the sample and the UV lamp was constant (60 mm) so that all the samples were treated under the identical conditions. Figure S1f illustrates the UV reactor system. The solution was irradiated for 5, 10, 15, and 20 min. After irradiation, the solutions were placed in closed containers with a humid atmosphere for 12 h. Moisture plays the role of antisolvent to coagulate cellulose. Finally, the hydrogels were subjected to a series of washes with distilled water to remove residues from the solvent system. The cured hydrogels were kept in humid atmospheres until evaluation.

### 2.5. Characterization

The characterization of the functional groups was carried out using a Fourier-transform infrared spectrometer (GX-FTIR, PerkinElmer, Waltham, MA, USA). For this analysis, the gel samples irradiated with UV light and the blank (untreated gel) were dehydrated in an oven for 24 h at 60 °C. Spectra were obtained by reflectance with an ATR accessory (Attenuated Total Reflectance, PerkinElmer, Waltham, MA, USA); each spectrum corresponds to the average of 30 scans with a resolution of 4 cm^−1^ in the range of 400 to 4000 cm^−1^.

For thermal stability analysis, dehydrated gels were employed. A thermal analyzer (SDT Q600, TA Instruments, New Castle, DE, USA) with a sensitivity of 0.1 μg was used. Measurements were performed with 10 mg of samples, heating from room temperature to 800 °C under an air atmosphere at a heating rate of 10 °C min^−1^.

Additionally, to study the effect of UV radiation and the reconstitution process on the crystalline structure of cellulose, an X-ray diffractometer (X’Pert PRO RX04, Malvern Panalytical, Almelo, Overijssel, The Netherlands) was used. For the analysis, the experimental conditions were: scan range from 5 to 60°, a step size of 0.0330 and 60 s for the counting time.

For the analyses by rotational rheometry (viscoelasticity) and dynamic mechanical analysis (stress–strain), the hydrogels treated with UV radiation and an untreated blank were used. For the former, hydrogel samples 10 mm in diameter and 1.5 mm in thickness were analyzed, while for the second, samples of 25 mm × 5 mm × 1.5 mm (length, width, thickness) were analyzed.

The viscoelasticity of the samples was accomplished by small-amplitude oscillatory shear (SAOS) using a rotational rheometer (Physica MCR 501, Anton Paar, Graz, Styria, Austria) employing the parallel plate geometry, with a plate diameter of 25 mm, a gap of 1 mm, and 25 °C. Strain sweeps were scanned to define the structural strength and the linear viscoelastic regime of the samples. Dynamic frequency sweep tests were achieved from 0.01 to 100 Hz and 0.1% strain, which is in the linear viscoelastic regime, to evaluate the structure and the viscoelastic properties of a sample as a timescale function.

The stress–strain tests were performed in a dynamic mechanical analyzer (RSAIII, TA Instruments), which has a transducer with a maximum applicable force capacity of 3.5 kg. In this equipment, a static deformation test was carried out in the tension mode, applying a deformation speed of 0.001 mm s^−1^ at 25 °C. In addition, dynamic strain scans were performed at a frequency of 1 Hz from an initial strain of 0.01% to a final strain of 1.0%. The strain increases were 0.02%, at 25 °C.

Morphology samples were obtained with a field emission scanning electron microscope (FESEM JEOL JSM-7401F, JEOL, Akishima, Japan) at a magnification of 2500 and 10,000.

## 3. Results and Discussion

**Figure 1:**
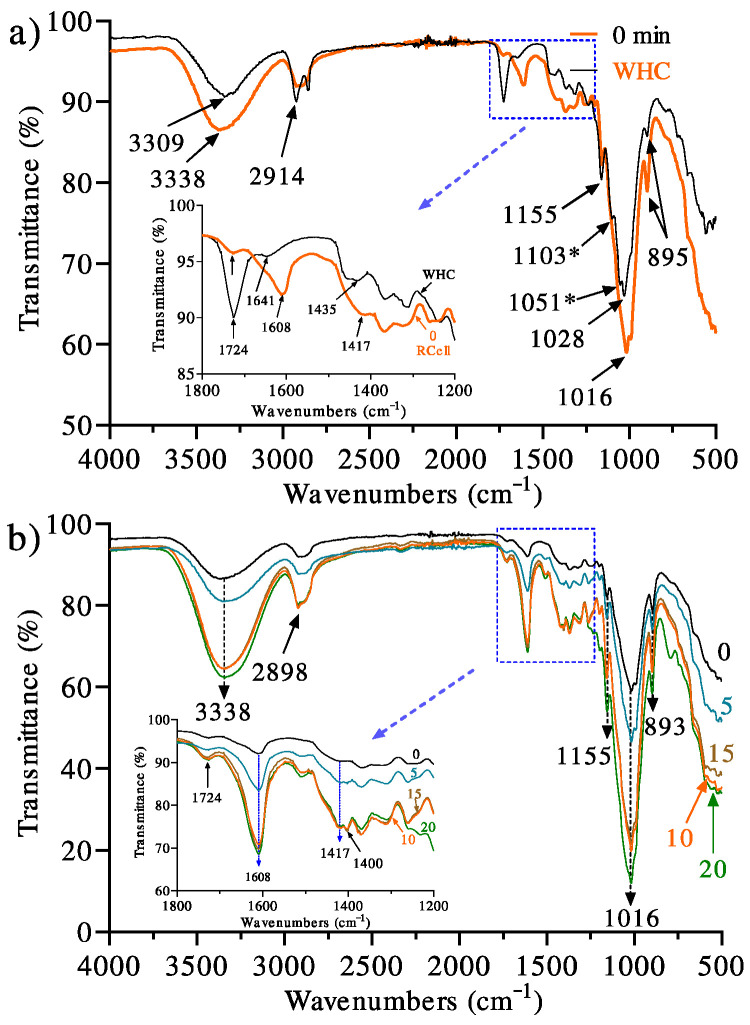
Infrared spectra of (**a**) cellulose fiber extracted from the wheat husk (WHC) and regenerated cellulose (0 min), without exposure to UV radiation, from solution in the DMAc/LiCl solvent system and (**b**) exposure time effect of UV radiation (0, 5, 10, 15, and 20 min) on regenerated cellulose from solution in the DMAc/LiCl solvent system. * Only present in 0 min.

Figure 1a shows the infrared spectrum of cellulose fibers extracted from the wheat husk (WHC) highlighting the absorption at 3309 cm^−1^ attributed to the stretching of the OH group. The peaks at 2914 and 2848 cm^−1^ correspond to the asymmetric and symmetric vibrations of the CH_2_ group, respectively. The signal at 1724 cm^−1^ (inset) attributed to the carbonyl (C=O) of ester groups of the hemicelluloses and the remnant of the lignin that was not completely extracted. Near this signal, a small absorption at 1641 cm^−1^ is observed, which is commonly associated with absorbed water [28,29]. The signal at 1435 cm^−1^ assigned to the scissoring bending of the CH_2_ group is strong for crystalline cellulose I. The absorption at 1155 cm^−1^ corresponds to the asymmetric vibration of the C–O–C bond and the bending of the OH bond of the C-OH group. On the other hand, the signals at 1103, 1051, and 1016 cm^−1^ are associated, respectively, with the glucose ring’s asymmetric stretching and stretching of the C-O bonds. In addition, the absorption at 895 cm^−1^ is representative of the stretching of the β (1 → 4) glycosidic linkage (C–O–C). When this band is sharp, it is related to cellulose II and amorphous cellulose. The absorptions reported here appropriately coincide with other reports in the literature [30,31].

After the dissolution of the cellulose in the DMAc-LiCl solvent system and regeneration, some differences were observed. It is important to note that this cellulose sample (0 min) was thoroughly washed with water to extract the DMAc-LiCl mixture, left dry, and no UV radiation was applied; i.e., changes in the infrared spectrum correspond only to the effect of regeneration. Thus, it is observed that the absorptions at 3309, 1724, 1435, and 1028 cm^−1^ described for WHC suffered displacements at 3338, 1608, 1417, and 1016 cm^−1^, respectively. All indicated signals are related to oxygen-containing groups, which are susceptible to hydrogen bonding. It is also appropriate to point out that the literature does not indicate any chemical reaction of cellulose with the solvent system. 

Consequently, the changes in the spectrum are not associated with chemical reactions but only with hydrogen bonding modification during regeneration. However, it should also be noted that the signals corresponding to the glycosidic ring asymmetric stretching (1103 cm^−1^) and stretching of the C–O bonds (1051 cm^−1^) are not observed in 0 min, which indicates that some interactions of the glycosidic ring are lost and are most likely related to the cellulose crystalline structure that was lost in regeneration. The lack of these absorptions has also been reported for cellulose regenerated from a recycled newspaper [32,33] and other celluloses [34,35]. Therefore, the idea that the loss of crystallinity causes the aforementioned effects is proposed.

Furthermore, the signal at 1435 cm^−1^, indicative of crystalline cellulose (cellulose I) of WHC, shifted to 1417 cm^−1^ in 0 min (no UV treatment), typical for amorphous cellulose II. An equivalent displacement was reported by other authors, who analyzed the effect of cellulose regeneration from different solvents; said shift was attributed to the loss of crystallinity in 0 min [30] and the elimination of intramolecular hydrogen bonding [36]. This result is consistent with the X-ray analysis that will be discussed later. 

Figure 1b shows the infrared spectra of dehydrated hydrogels treated with UV irradiation. As noted, some significant differences are observed. As for the celluloses irradiated at exposure times of 5, 10, 15, and 20 min, the progressive increase in the intensities of the absorptions at 3338, 1608, and 1016 cm^−1^ are indicative of the effects launched by the UV radiation. In particular, we consider that a combined effect (degradation-crosslinking) is the cause of these results in such a way that the abundance of functional groups and bonds involved with these absorptions increases with the irradiation time.

Although the mechanism of cellulose photooxidation has not been fully established, in general, it has accepted the formation of peroxyl radicals due to treatment with UV radiation, and these radicals the formation of hydroperoxide groups, which, through constant UV irradiation and their high energy level, break homolytically to form highly reactive hydroxyl groups, which initiate a dehydrogenation process in the cellulose chain, increasing the respective signals of –OH groups, and C–O, C–H, and C–OH bonds [37].

It is worth noting that, in this work, the irradiation of the cellulose in solution has a substantial effect that allows for the free passage of UV radiation towards all the molecules in the bulk because the system is transparent. However, when cellulose is in the solid-state, this does not occur, as reported by Yang and Freeman [38], who found that the distribution of chemical species was not identical on the surface and in bulk in cotton textile samples irradiated with UV light. Kaczmarek et al. [39] and Ołdak et al. [40] reported, respectively, after 10 and 100 h exposure to UV radiation, no significant changes in cellulose stability. Both groups attributed the photostability to the high purity of their celluloses used; however, other studies have reported that the effect of radiation on the surface and the bulk are different. Zapolskii [41] studied the photooxidation of cellulose with UV radiation in the presence of air, irradiating the sample for 20 h with a quartz lamp, observing an increase in signal intensities in the infrared spectrum corresponding to the stretching of C-O bonds and a reduction in the intensity of signals representative of the vibrations of the C-O and C-C bonds of the glucose unit residues, which was attributed to the breaking of the glycosidic bonds during the degradation process and the oxidation of the primary alcohol groups due to the radiation action. On the other hand, Yang and Freeman [38] obtained similar results when exposing cotton cellulose in fabric samples to a UV radiation of 254 nm at room temperature, showing an increase in the carbonyl group signals while increasing the radiation exposure time. The results showed that the main photooxidation product in the samples was carboxylic acids [38]. In the present research, the peak height of carbonyl groups in cellulose membrane samples is extremely weak, which could be related to a much shorter time of exposure to UV radiation in comparison to the mentioned works.

Kaczmarek et al. [39] reported that the photodegradation with UV radiation, at 254 nm and 25 °C, on polypropylene/cellulose composites showed a greater improvement than that which occurred in its pure component because the cellulose sample presented high purity. It was observed that even in composites with low cellulose content (5–15%), the photooxidation process led to the formation of carbonyl, hydroxyl, and hydroperoxide groups. This was attributed to the increase in the amorphous part of the polypropylene during the mixing process, which facilitates the access of oxygen to the polymer chains and increased the flexibility of the macromolecules, resulting in a more efficient oxidation process. Similarly, regenerated cellulose from waste has low crystallinity, allowing for the stabilization of oxygen during the photooxidation process. In addition to the fact that it carries impurities that contain chromophore groups capable of enhancing the photooxidation process causing the formation of and increase in the functional groups content. Hence, the increase in signal intensities in a shorter time of exposure to UV radiation could be explained.

### 3.1. Effect of UV Radiation on the Thermal Stability

**Figure 2:**
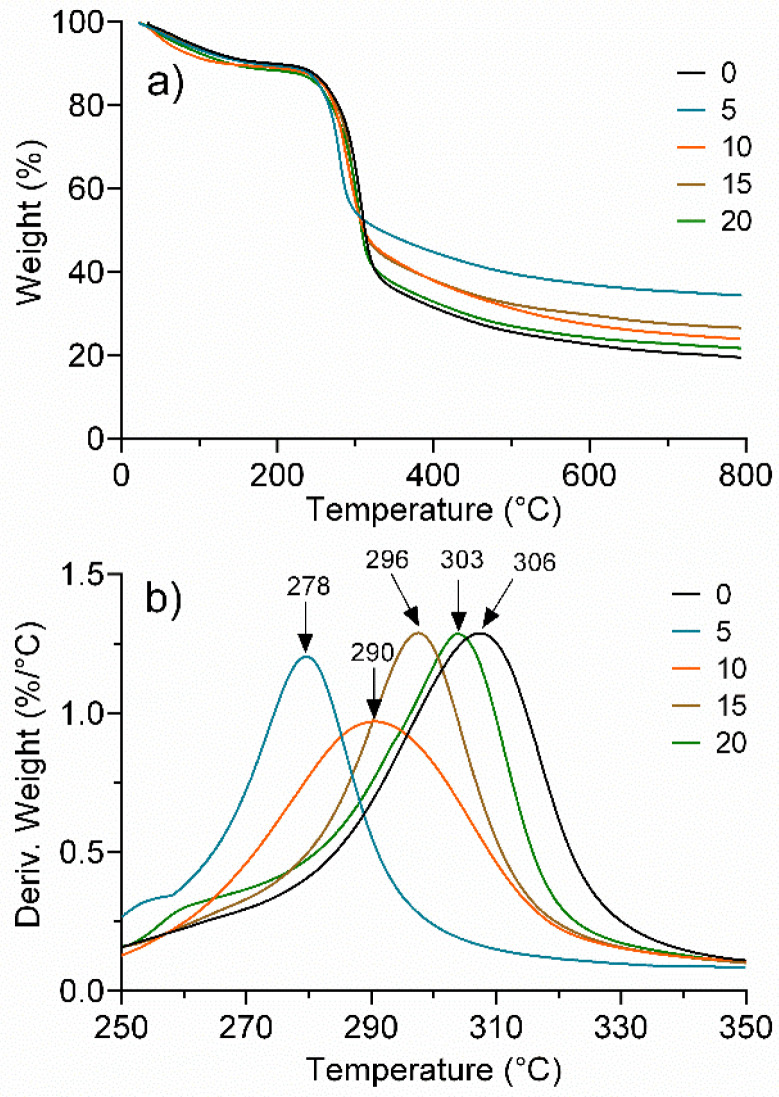
Effect of UV radiation time (0, 5, 10, 15, and 20 min) on the thermal stability of regenerated cellulose. (**a**) TG and (**b**) DTG.

Figure 2a shows the TGA traces of regenerated cellulose exposed to UV radiation at different times. Thermograms show a three-step degradation process, where the first drop corresponds to approximately 12–15% weight loss between 40 and 150 °C, associated with the evaporation of traces of water or dehydration. The second drop corresponds to a 55–60% loss in weight in a range of 200 to 300 °C, attributed to the decomposition of the cellulose main chain [42]. The third transition, corresponding to a 25–30% weight loss, in the range of 320 to 800 °C, is attributed to the presence of the remaining carbon [43]. It should be noted that from TGA traces, an essentially identical behavior was observed in all the samples without any significant difference between them regardless of the UV treatment time.

The DTG analysis, Figure 2b, in contrast, provided considerable evidence of the effect of UV irradiation time. In particular, the peak between 250 and 350 °C does indicate a significant difference for the treatments. It is observed that for 0 min, which was not irradiated, the maximum peak indicates the highest thermal decomposition rate, presenting the highest value (306 °C). However, with only 5 min of exposure, the peak decreased by 28 °C, which indicates a significant loss of thermal stability, and that is associated with a degradative process due to the effect of UV radiation. For the samples with longer exposure times, a progressive increase in the peak was observed, until, at 20 min of exposure (303 °C), the peak of 0 min was almost equalized. This behavior implies that after an initial photooxidative degradation process, a chemical crosslinking mechanism is initiated that compensates for the degradative effect and almost leads to the initial thermal stability.

### 3.2. Crystallinity

**Figure 3:**
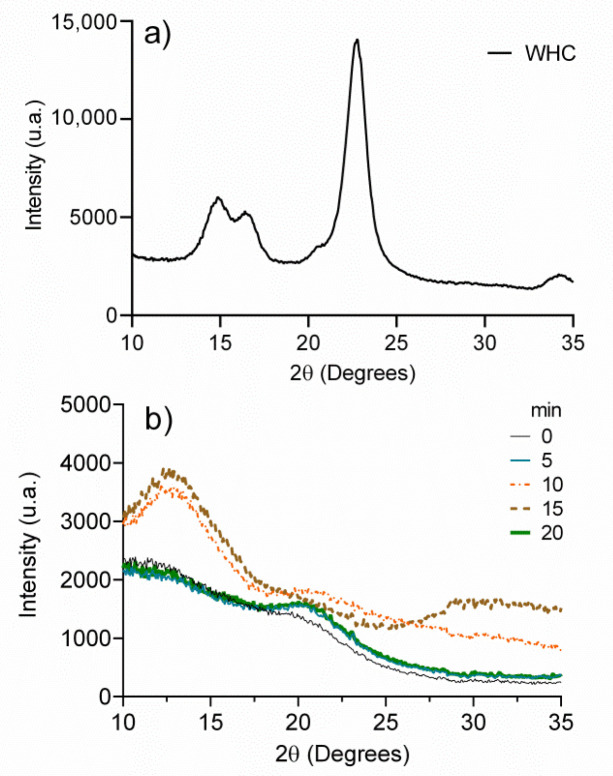
(**a**) X-ray spectrum of cellulose extracted from the wheat husk (WHC) and (**b**) X-ray spectra of regenerated celluloses at different exposure times to UV radiation.

To determine the effect of the exposure time of UV radiation on the crystallinity of the dehydrated hydrogels, X-ray diffractometry was used. Figure 3 illustrates the diffractograms of WHC and regenerated celluloses exposed to UV radiation (0, 5, 10, 15, and 20 min). As seen, the crystalline form of cellulose I of WHC (Figure 3a) shows diffraction peaks at 2θ = 14.87°, 16.40°, 22.73°, and 34.25° attributed to the planes (11′0), (110), (200), and (004), respectively [44]. On the other hand, the UV-treated, regenerated cellulose samples (Figure 3b) show amorphous signals at 2θ = 12.70° and 20.77° that are observed in more detail at 10 and 15 min of irradiation, which is representative of the amorphous structure of cellulose II, the signal at 2θ = 12.70° being characteristic of the plane (101) [45]. It should be noted that the sample without UV treatment (0 min) shows peaks corresponding to the amorphous region with weak intensity, which indicates a very low crystallinity of the regenerated cellulose. These results coincide with the observed in infrared spectroscopy, in which the displacement and absence of signals were related to the loss of the cellulose crystalline structure. The loss of crystallinity in cellulose after the regeneration process is normal, as evidenced by the extensive reported literature [32,34].

### 3.3. Rheology

Dynamic frequency sweep tests were performed to evaluate the structure and the viscoelastic properties (storage modulus (G′) and loss modulus (G″)) of the hydrogels as a function of timescale and UV treatment. 

A material is classified as a gel when the resulting G′ and G″ are frequency independent or they exhibited a weak frequency dependence. The curves of G′ and G″ often occur in the form of almost parallel straight lines throughout the entire frequency range, showing only a slight slope. If the curves of G′ and G″ present a slope less than 0.05, it is considered that they are relatively frequency independent [46]. Furthermore, the relationship between G′ and G″ can be used to describe the “viscoelastic character” of a sample; i.e., if G′ > G″, the elastic behavior dominates the viscous one, but if G″ > G′, the opposite is true [47]. 

**Figure 4:**
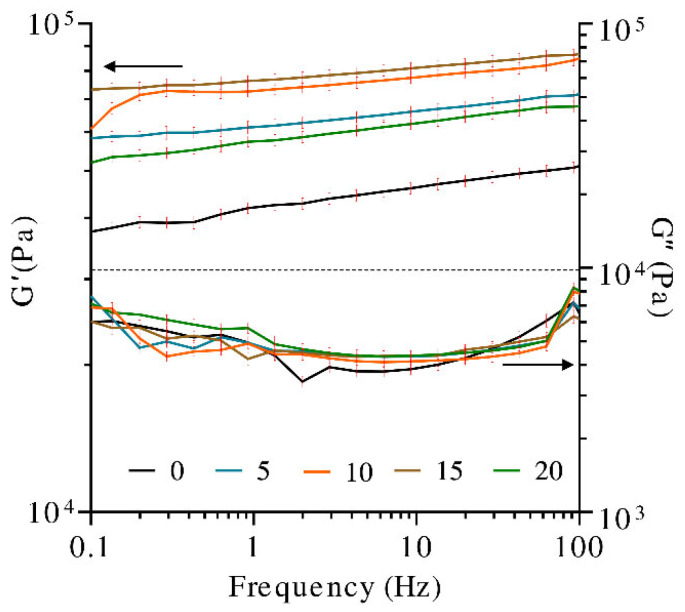
Dynamic frequency sweep tests (storage modulus (G′) and loss modulus (G″) in hydrogels at different exposure times to UV radiation).

As observed in Figure 4, the storage modulus (G′) was always higher than the loss modulus (G″), denoting that the solid-like behavior (elastic character) predominates over the liquid-like character. All of the hydrogels showed no crossing points and negligible dependence of the frequency on the storage and loss moduli since G′ curves presented slopes less than 0.05. There is a small decrease in the moduli, as the angular frequency decrease points to the existence of relaxation processes, which can be induced by the release of entrapped entanglements or the opening of ionic junction points. These phenomena are not very pronounced, and the gels mainly exhibit rubber-elastic properties [48].

Furthermore, the literature indicates that UV exposure time, up to a certain threshold, leads to a higher number of functional crosslinks and enhances the viscoelastic properties of the hydrogels. Longer exposure times allow for the photopolymerization reaction to proceed to completion [49].

In the present study, samples of hydrogel were irradiated at different times. The storage modulus (G′) of the hydrogels before and after exposure to UV light for 5, 10, 15, and 20 min was analyzed. It was observed that the sample with no UV treatment presented the lowest G′. Due to the increase in the exposure time to UV radiation, up to 15 min, the hydrogel strength, G′, was promoted. A higher degree of crosslinking in the covalently crosslinked polymeric network is obtained. The higher crosslinking degree caused the closer distance between the chain segments and made the movement of molecular chains more difficult, increasing the hydrogel stiffness [50]. Contrastingly, the G′ obtained with 20 min of the UV treatment was lower than that obtained with 5 min, indicating that the photocrosslinking effect on G′ decreases after 15 min of exposure to UV light. Photodegradation or intramolecular cycles (chain loops) may be occurring beyond 15 min, producing adverse effects in the hydrogel. It has been shown that the UV exposure time affects the resultant hydrogel network defects, which has a pronounced effect on the hydrogel modulus [51]. For example, chain entanglements cause an increase in crosslinking density within the polymer network, which in turn results in a higher modulus [52]. Alternatively, intramolecular cycles (chain loops) decrease crosslinking density, thus negatively affecting hydrogel modulus [49]. Significant differences have also been observed for gelatin hydrogels modified with nanocellulose, in which the application of gamma radiation has shown the generation of chemical crosslinking between the biopolymer molecules, reflected in G′ increments greater than 200% [53].

### 3.4. Stress-Strain Behavior

To accurately determine the mechanical properties of the hydrogels and to develop morphological relationships, their linear viscoelastic region was determined by DMA, employing a strain sweep test. Within this region, the material response is independent of the deformation magnitude, and the material structure is maintained intact. 

**Figure 5:**
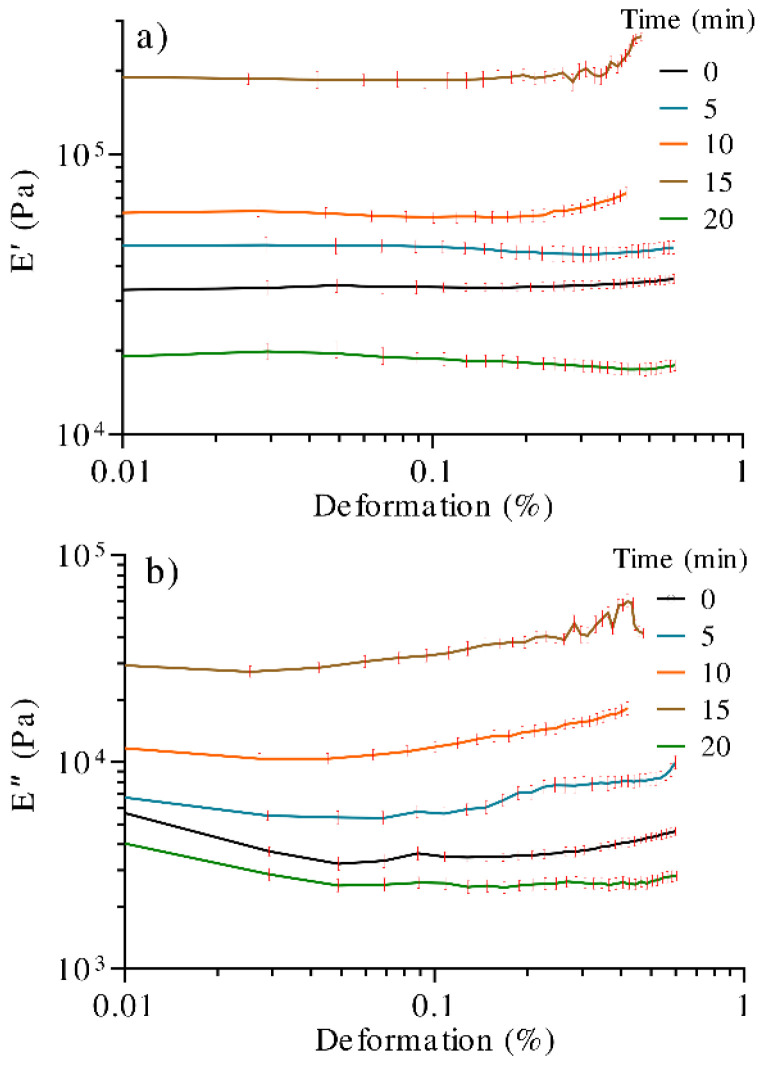
DMA curves of hydrogels treated with different times of UV radiation. (**a**) Storage modulus (E′) and (**b**) loss modulus (E″).

Figure 5a shows the relationship between the UV irradiation time and the storage modulus (E′). The overall trend indicates that E′ is modified with irradiation time, which has been attributed to the increase in the gel crosslinking density [54]. As noted, the hydrogel irradiated for 15 min showed the best result, close to 190,000 Pa, and a linear viscoelastic region that extends up to a deformation percentage of 0.32%. However, after this value, the storage modulus experiments an increment, meaning that the material shows a stiffening effect, which could be attributed to moisture loss. For this reason, it is difficult to determine the limit of the linear viscoelastic region of the samples. Following this, the 10, 5, and 0 min samples showed storage moduli in the range of 30,000–60,000 Pa, while the 20 min sample showed the lowest value. The loss modulus (E″), Figure 5b, showed the equivalent behavior as E′ respecting UV exposition time; however, E′ exhibited a more major increase than E″.

As observed, E′ > E″, which is representative of a reversible viscoelastic behavior (characteristic of a gel or solid), and that, in this case, the elastic behavior dominates the viscous one, showing rigidity in the samples.

**Figure 6:**
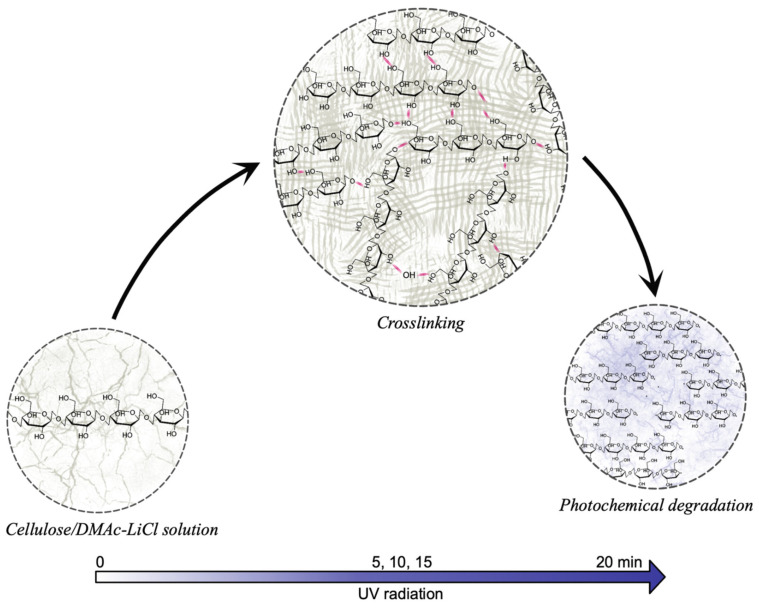
Effect of UV radiation on cellulose in DMAc/LiCl solution. At times greater than 20 min, the photochemical degradation exceeds the crosslinking reactions; this being reflected in the decrease in mechanical properties.

The increasing tendency in resistance to deformation respecting UV treatment in the hydrogel was related to the intermolecular interaction between adjacent cellulose chains, resulting in a crosslinking process, as well as the formation of low-molecular-weight polymeric radicals, which, as reported [55], take part in the photodegradation. A proposed representative model of the process is shown in Figure 6.

The photooxidation of cellulose and its derivatives has been studied for over a hundred years, as pointed out by Hon in 1976 [56]. Previous studies have emphasized the presence of oxygen as an essential condition in the photooxidation mechanism since it plays a vital role in the formation of free radicals. According to these models, there are a variety of labile points of photooxidation reactions, which include glycosidic bonds, OH groups, and H, from which free radicals are formed. The products of photooxidation, due to the effect of electronic rearrangement in the glucose ring, range from the breakdown of the glycosidic group, the formation of carbonyl groups, to the ring-opening. Figure S1 illustrates the micrographs treated with UV radiation (5, 10, 15, and 20 min) and without radiation (0 min). Compared with the 0 min, there are changes in the morphology of the samples, suggesting that UV treatment not only causes degradation, but also structural differences. For example, the sample at 15 min, clearly shows layers in one direction, suggesting that this time of radiation causes a packing of layers coinciding with the results of the mechanical property. On the contrary, at 20 min, no defined structure is shown, and therefore decrements in the modules were observed.

## 4. Conclusions

The effect of UV irradiation time on cellulose hydrogels was studied. Cellulose was isolated from the wheat husk and dissolved in *N,N*-dimethylacetamide/LiCl solvent. The different characterization techniques showed significant effects as a function of irradiation time, which was associated with crosslinking reactions. Notable differences were also determined in regenerated cellulose regarding isolated cellulose, mainly in the loss of crystallinity (cellulose I phase). According to DMA, with 15 min of UV treatment, E′ showed the best mechanical performance (200,000 Pa), compared to 0 min (33,000 Pa) and 20 min (20,000 Pa) UV treatment, showing a difference of 6 and 10 times, respectively. For the first case, the difference was associated with chemical crosslinking, while for the latter, the decrease was attributed to photodegradation. It should be noted that rotational rheometry showed an equivalent behavior. Finally, it can be concluded that UV radiation treatment demonstrated to be an efficient method to improve the mechanical properties of cellulose hydrogels. In this study, 15 min of UV treatment provided the best results; up to this point, the chemical crosslinking produces the greatest contribution to the hydrogel mechanical properties, while at a longer time, the photochemical degradation predominates, causing the opposite effect.

## Data Availability

Not applicable.

## Supplementary Materials

The following are available online at https://www.mdpi.com/article/10.3390/polym13142342/s1, Figure S1: FESEM Micrographs and reactor diagram for cellulose curing. (a) 0 min; (b) 5min; (c) 10 min; (d) 15 min; (e) 20 min; and (f) reactor consisting of a UV lamp, camera obscura and sample holder.

## References

[1] Kumar R., Sharma R.K., Singh A.P. (2018). Grafted cellulose: A bio-based polymer for durable applications. Polym. Bull..

[2] Gupta P.K., Raghunath S.S., Prasanna D.V., Venkat P., Shree V., Chithananthan C., Choudhary S., Surender K., Geetha K. (2019). An Update on Overview of Cellulose, Its Structure and Applications. Cellulose.

[3] Cai J., Zhang L. (2006). Unique Gelation Behavior of Cellulose in NaOH/Urea Aqueous Solution. Biomacromolecules.

[4] Zhou J., Zhang L., Cai J. (2004). Behavior of cellulose in NaOH/Urea aqueous solution characterized by light scattering and viscometry. J. Polym. Sci. Part B Polym. Phys..

[5] Pinkert A., Marsh K.N., Pang S., Staiger M. (2009). Ionic Liquids and Their Interaction with Cellulose. Chem. Rev..

[6] Kosan B., Michels C., Meister F. (2008). Dissolution and forming of cellulose with ionic liquids. Cellulose.

[7] McCormick C.L., Callais P.A. (1986). Derivatization of cellulose in lithium chloride and N-N-dimethylacetamide solutions. Am. Chem. Soc. Polym. Prepr. Div. Polym. Chem..

[8] Zhang C., Liu R., Xiang J., Kang H., Liu Z., Huang Y. (2014). Dissolution Mechanism of Cellulose in N,N-Dimethylacetamide/Lithium Chloride: Revisiting through Molecular Interactions. J. Phys. Chem. B.

[9] McCormick C.L. (1981). Novel Cellulose Solutions.

[10] Sayyed A., Deshmukh N.A., Pinjari D.V. (2019). A critical review of manufacturing processes used in regenerated cellulosic fibres: Viscose, cellulose acetate, cuprammonium, LiCl/DMAc, ionic liquids, and NMMO based lyocell. Cellulose.

[11] Ono Y., Furihata K., Isobe N., Saito T., Isogai A. (2018). Solution-state structures of the cellulose model pullulan in lithium chloride/N,N-dimethylacetamide. Int. J. Biol. Macromol..

[12] Chang C., Duan B., Cai J., Zhang L. (2010). Superabsorbent hydrogels based on cellulose for smart swelling and controllable delivery. Eur. Polym. J..

[13] Fu L.-H., Qi C., Ma M.-G., Wan P. (2019). Multifunctional cellulose-based hydrogels for biomedical applications. J. Mater. Chem. B.

[14] Anseth K.S., Bowman C., Brannon-Peppas L. (1996). Mechanical properties of hydrogels and their experimental determination. Biomaterials.

[15] Pan J., Jin Y., Lai S., Shi L., Fan W., Shen Y. (2019). An antibacterial hydrogel with desirable mechanical, self-healing and recyclable properties based on triple-physical crosslinking. Chem. Eng. J..

[16] Wang Y., Wang J., Yuan Z., Han H., Li T., Li L., Guo X. (2017). Chitosan cross-linked poly(acrylic acid) hydrogels: Drug release control and mechanism. Colloids Surf. B Biointerfaces.

[17] Liang H.-C., Chang W.-H., Liang H.-F., Lee M.-H., Sung H.-W. (2004). Crosslinking structures of gelatin hydrogels crosslinked with genipin or a water-soluble carbodiimide. J. Appl. Polym. Sci..

[18] Demitri C., Del Sole R., Scalera F., Sannino A., Vasapollo G., Maffezzoli A., Ambrosio L., Nicolais L. (2008). Novel superabsorbent cellulose-based hydrogels crosslinked with citric acid. J. Appl. Polym. Sci..

[19] Ennker J., Ennker I.C., Schoon D., Schoon H.A., Dörge S., Meissler M., Rimpler M., Hetzer R. (1994). The impact of gelatin-resorcinol glue on aortic tissue: A histomorphologic evaluation. J. Vasc. Surg..

[20] Ishak W.H.W., Jia O.Y., Ahmad I. (2020). pH-Responsive Gamma-Irradiated Poly(Acrylic Acid)-Cellulose-Nanocrystal-Reinforced Hydrogels. Polymers.

[21] Sionkowska A., Wisniewski M., Skopinska J., Poggi G., Marsano E., Maxwell C., Wess T. (2006). Thermal and mechanical properties of UV irradiated collagen/chitosan thin films. Polym. Degrad. Stab..

[22] Wach R.A., Mitomo H., Nagasawa N., Yoshii F. (2003). Radiation crosslinking of methylcellulose and hydroxyethylcellulose in concentrated aqueous solutions. Nucl. Instrum. Methods Phys. Res. Sect. B Beam Interact. Mater. Atoms.

[23] Yang X., Ku T.-H., Biswas S.K., Yano H., Abe K. (2019). UV grafting: Surface modification of cellulose nanofibers without the use of organic solvents. Green Chem..

[24] Nair J.R., Gerbaldi C., Chiappone A., Zeno E., Bongiovanni R., Bodoardo S., Penazzi N. (2009). UV-cured polymer electrolyte membranes for Li-cells: Improved mechanical properties by a novel cellulose reinforcement. Electrochem. Commun..

[25] Zhang X., Xu L., Huang X., Wei S., Zhai M. (2012). Structural study and preliminary biological evaluation on the collagen hydrogel crosslinked by γ-irradiation. J. Biomed. Mater. Res. Part A.

[26] Snelders J., Dornez E., Benjelloun-Mlayah B., Huijgen W.J., de Wild P.J., Gosselink R.J., Gerritsma J., Courtin C.M. (2014). Biorefining of wheat straw using an acetic and formic acid based organosolv fractionation process. Bioresour. Technol..

[27] McCormick C.L., Callais P.A., Hutchinson B.H. (1985). Solution studies of cellulose in lithium chloride and N,N-dimethylacetamide. Macromolecules.

[28] Lu P., Hsieh Y.-L. (2010). Preparation and properties of cellulose nanocrystals: Rods, spheres, and network. Carbohydr. Polym..

[29] Ciolacu D., Ciolacu F., Popa V.I. (2011). Amorphous cellulose—Structure and characterization. Cellul. Chem. Technol..

[30] Kuo C.-H., Lee C.-K. (2009). Enhancement of enzymatic saccharification of cellulose by cellulose dissolution pretreatments. Carbohydr. Polym..

[31] De Oliveira J.P., Bruni G.P., Lima K.O., El Halal S.L.M., DA Rosa G.S., Dias A.R.G., Zavareze E.D.R. (2017). Cellulose fibers extracted from rice and oat husks and their application in hydrogel. Food Chem..

[32] Mohamed M.A., Salleh W.W., Jaafar J., Ismail A.F., Mutalib M.A., Sani N., Asri S.M., Ong C.S. (2016). Physicochemical characteristic of regenerated cellulose/N-doped TiO_2_ nanocomposite membrane fabricated from recycled newspaper with photocatalytic activity under UV and visible light irradiation. Chem. Eng. J..

[33] Mohamed M.A., Salleh W., Jaafar J., Ismail A.F., Mutalib M.A., Jamil S.M. (2015). Incorporation of N-doped TiO_2_ nanorods in regenerated cellulose thin films fabricated from recycled newspaper as a green portable photocatalyst. Carbohydr. Polym..

[34] Yan L., Gao Z. (2008). Dissolving of cellulose in PEG/NaOH aqueous solution. Cellulose.

[35] Liu S., Zhang L. (2008). Effects of polymer concentration and coagulation temperature on the properties of regenerated cellulose films prepared from LiOH/urea solution. Cellulose.

[36] Zhang H., Wu J., Zhang J., He J. (2005). 1-Allyl-3-methylimidazolium Chloride Room Temperature Ionic Liquid: A New and Powerful Nonderivatizing Solvent for Cellulose. Macromolecules.

[37] Malešič J., Kolar J., Strlic M., Kočar D., Fromageot D., Lemaire J., Haillant O. (2005). Photo-induced degradation of cellulose. Polym. Degrad. Stab..

[38] Yang C.Q., Freeman J.M. (1991). Photo-Oxidation of Cotton Cellulose Studied by FT-IR Photoacoustic Spectroscopy. Appl. Spectrosc..

[39] Kaczmarek H., Ołdak D., Malanowski P., Chaberska H. (2005). Effect of short wavelength UV-irradiation on ageing of polypropylene/cellulose compositions. Polym. Degrad. Stab..

[40] Ołdak D., Kaczmarek H., Buffeteau T., Sourisseau C. (2005). Photo- and Bio-Degradation Processes in Polyethylene, Cellulose and their Blends Studied by ATR-FTIR and Raman Spectroscopies. J. Mater. Sci..

[41] Zapolskii O. (1963). Photo-oxidation of cellulose. Polym. Sci. U.S.S.R..

[42] Yeng L.C., Wahit M.U., Othman N. (2015). Thermal and flexural properties of regenerated cellulose(RC)/poly(3- hydroxybutyr-ate)(PHB) biocomposites. J. Teknol..

[43] Bengtsson A., Bengtsson J., Sedin M., Sjöholm E. (2019). Carbon Fibers from Lignin-Cellulose Precursors: Effect of Stabilization Conditions. ACS Sustain. Chem. Eng..

[44] Gong J., Li J., Xu J., Xiang Z., Mo L. (2017). Research on cellulose nanocrystals produced from cellulose sources with various polymorphs. RSC Adv..

[45] Ford E.N.J., Mendon S.K., Thames S.F., Rawlins J.W. (2010). X-ray Diffraction of Cotton Treated with Neutralized Vegetable Oil-based Macromolecular Crosslinkers. J. Eng. Fibers Fabr..

[46] Nicole M., Caimeng Z., Eric K., Yufei H. (2014). Salt and Acid-Induced Soft Tofu-Type Gels: Rheology, Structure and Fractal Analysis of Viscoelastic Properties as a Function of Coagulant Concentration. Int. J. Food Eng..

[47] Mezger T. (2020). The Rheology Handbook.

[48] Leick S., Henning S., Degen P., Suter D., Rehage H. (2010). Deformation of liquid-filled calcium alginate capsules in a spinning drop apparatus. Phys. Chem. Chem. Phys..

[49] Sheth S., Jain E., Karadaghy A., Syed S., Stevenson H., Zustiak S.P. (2017). UV Dose Governs UV-Polymerized Polyacrylamide Hydrogel Modulus. Int. J. Polym. Sci..

[50] Huang M., Hou Y., Li Y., Wang D., Zhang L. (2017). High performances of dual network PVA hydrogel modified by PVP using borax as the structure-forming accelerator. Des. Monomers Polym..

[51] Baselga J., Llorente M., Hernández-Fuentes I., Piérola I. (1989). Network defects in polyacrylamide gels. Eur. Polym. J..

[52] Chassé W., Lang M., Sommer J.-U., Saalwächter K. (2012). Cross-Link Density Estimation of PDMS Networks with Precise Consideration of Networks Defects. Macromolecules.

[53] Ishak W.H.W., Ahmad I., Ramli S., Amin M.C.I.M. (2018). Gamma Irradiation-Assisted Synthesis of Cellulose Nanocrystal-Reinforced Gelatin Hydrogels. Nanomaterials.

[54] Lin S., Gu L. (2015). Influence of Crosslink Density and Stiffness on Mechanical Properties of Type I Collagen Gel. Materials.

[55] Yousif E., Haddad R. (2013). Photodegradation and photostabilization of polymers, especially polystyrene: Review. SpringerPlus.

[56] Hon N.-S. (1976). Formation of free radicals in photoirradiated cellulose. VIII. Mechanisms. J. Polym. Sci. Polym. Chem. Ed..

