# Supplementary material for: Curing of Cellulose Hydrogels by UV Radiation for Mechanical Reinforcement
Source: Polymers (Basel). 2021 Jul 17;13(14):2342. doi: 10.3390/polym13142342 (PMC8309531; doi:10.3390/polym13142342)
Supplement: Supplementary file 1 [file polymers-13-02342-s001.zip › polymers-1284390-supplementary.pdf]

Material supplementary

Curing of cellulose hydrogels by UV radiation for mechanical reinforcement

Rodybeth Cruz-Medina[1], Daniel A. Ayala-Hernández[1], Alejandro Vega-Rios[1], Erika I. López-Martínez[1], Mónica E. Mendoza-Duarte[1], Anayansi Estrada-Monje[2] and E. Armando Zaragoza-Contreras[1,*]

[1] Department of Engineering and Materials Chemistry, Centro de Investigación en Materiales Avanzados, SC, Miguel de Cervantes No. 120, Complejo de Industrial Chihuahua, Chihuahua, Chih. CP 31136.
[2] Centro de Innovación Aplicada en Tecnologías Competitivas, AC, Calle Omega No. 201, Industrial Delta, León, Guanajuato. CP 37545.

* Correspondence: armando.zaragoza@cimav.edu.mx; Tel.: (+52 614 4394811)

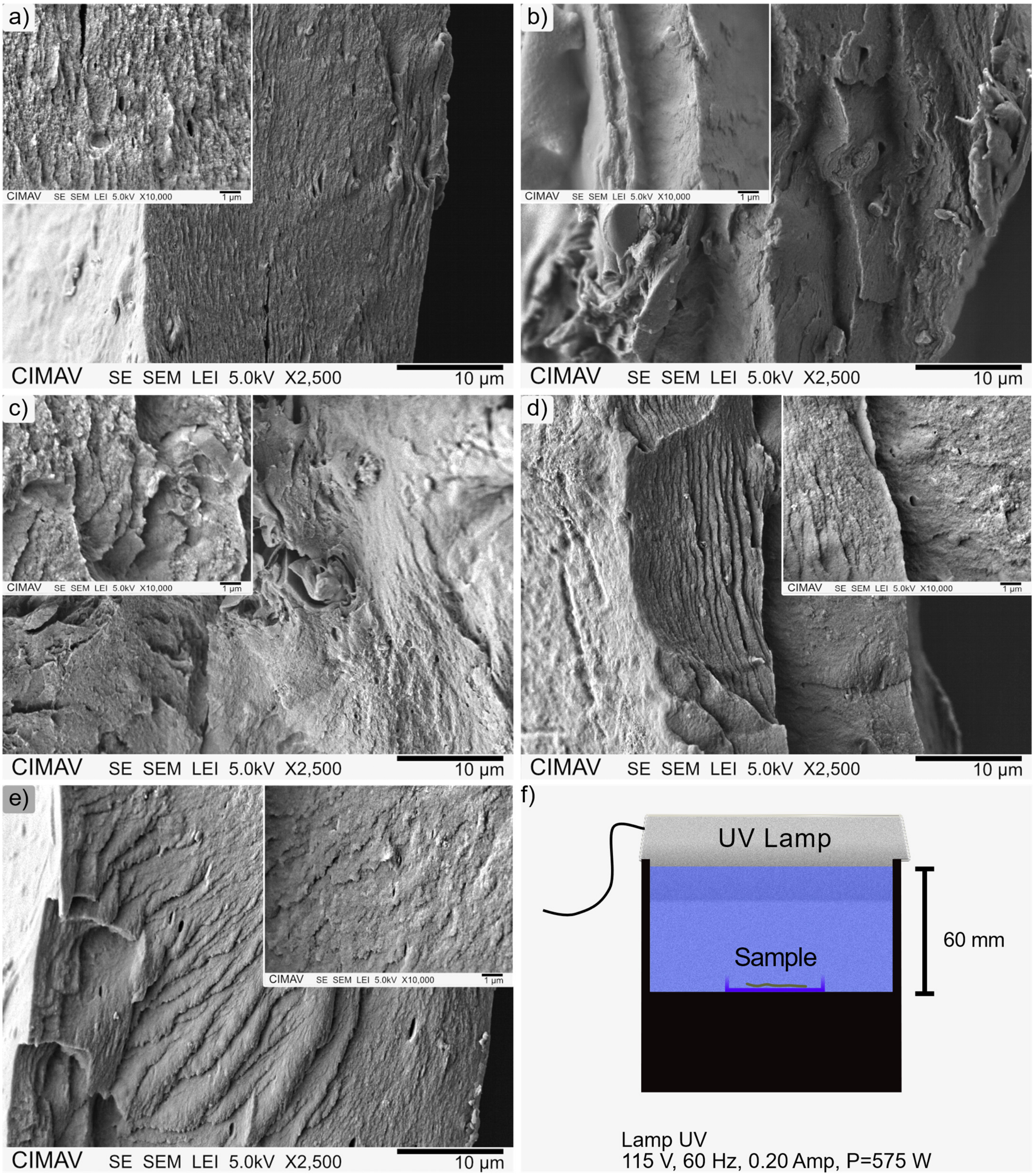

Figure S1. FESEM Micrographs and reactor diagram for cellulose curing. (**a**) 0 min; (**b**) 5min; (**c**) 10 min; (**d**) 15 min; (**e**) 20 min; and (**f**) reactor consisting of a UV lamp, camera obscura and sample holder.
